# Bioinformatic analysis of type III CRISPR systems reveals key properties and new effector families

**DOI:** 10.1093/nar/gkae462

**Published:** 2024-05-29

**Authors:** Ville Hoikkala, Shirley Graham, Malcolm F White

**Affiliations:** School of Biology, University of St Andrews, St Andrews KY16 9ST, UK; Department of Biological and Environmental Science, University of Jyväskylä, Jyväskylä, Finland; School of Biology, University of St Andrews, St Andrews KY16 9ST, UK; School of Biology, University of St Andrews, St Andrews KY16 9ST, UK

## Abstract

Recognition of RNA from invading mobile genetic elements (MGE) prompts type III CRISPR systems to activate an HD nuclease domain and/or a nucleotide cyclase domain in the Cas10 subunit, eliciting an immune response. The cyclase domain can generate a range of nucleotide second messengers, which in turn activate a diverse family of ancillary effector proteins. These provide immunity by non-specific degradation of host and MGE nucleic acids or proteins, perturbation of membrane potentials, transcriptional responses, or the arrest of translation. The wide range of nucleotide activators and downstream effectors generates a complex picture that is gradually being resolved. Here, we carry out a global bioinformatic analysis of type III CRISPR loci in prokaryotic genomes, defining the relationships of Cas10 proteins and their ancillary effectors. Our study reveals that cyclic tetra-adenylate is by far the most common signalling molecule used and that many loci have multiple effectors. These typically share the same activator and may work synergistically to combat MGE. We propose four new candidate effector protein families and confirm experimentally that the Csm6-2 protein, a highly diverged, fused Csm6 effector, is a ribonuclease activated by cyclic hexa-adenylate.

## Graphical Abstract

**Graphical Abstract:**
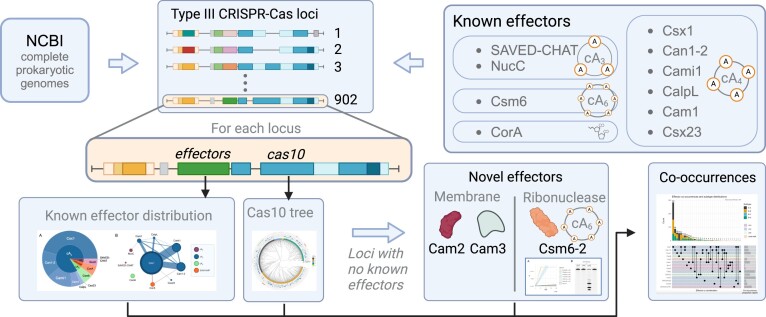

## Introduction

CRISPR-Cas is an adaptive prokaryotic immune system that integrates fragments of invading nucleic sequences, usually from viruses, as spacers into a chromosomal CRISPR array (1). Upon subsequent infection, transcribed spacers in the form of CRISPR RNA guide CRISPR associated (Cas) interference proteins to a complementary site on the invading nucleic acid. In type III CRISPR systems, this interference response is facilitated by a multi-protein complex, hallmarked by the Cas10 protein (2). Once type III effectors bind the invading RNA, Cas10 provides an immune response by activating two potential enzymatic activities: an N-terminal HD nuclease domain that cleaves ssDNA non-specifically (3–5) and a PALM polymerase domain that synthesizes cyclic oligoadenylate (cOA) signalling molecules (6,7). Within the Cas10 family, cyclase activity is more common than nuclease activity, but the two active sites can co-occur. cOA signalling molecules, which can range from cyclic tri- to hexa-adenylate (cA_3_, cA_4_, cA_6_), bind and activate ancillary effectors which are often encoded by genes in the same CRISPR-Cas operon (reviewed in (8,9)). *In vitro*, type III CRISPR systems typically generate a range of cOA species (6,10–13), but the range and relative abundance can differ quite markedly *in vivo* (14). Recently, a type III-B system that conjugates *S*-adenosyl methionine and ATP to make the second messenger SAM-AMP has been described (15), increasing the diversity further.

Ten diverse type III CRISPR ancillary effector families have been characterized biochemically. Each is activated by one specific signalling molecule. We will use the following definitions for our study:

**Csx1** – this encompasses a large and diverse family whose members have a CARF domain fused to a HEPN ribonuclease domain. These dimeric proteins bind cA_4_, activating the HEPN domain for non-specific mRNA degradation (16–20). We have merged some cA_4_-dependent proteins previously annotated as Csm6 proteins (21,22) into this group.

**Csm6** – we define this family as dimeric CARF-HEPN proteins activated by cA_6_ (14,23,24).

**Can1-2** – this includes the Can1 and Can2/Card1 family of cA_4_ activated CARF-nuclease effectors, which degrade both DNA and RNA (25–27). Can1 is a monomer and Can2 a dimer. In this study, we treat them as one effector class.

**Cami1** – the recently described Cami1 family are dimeric, cA_4_ activated proteins with a CARF domain fused to a RelE family nuclease. On activation, they cleave mRNA at the ribosomal A-site to shut down translation (28).

**CalpL** – the CalpL family are monomers with a SAVED domain for cA_4_ recognition fused to a Lon-family protease. On activation, CalpL self-associates and cleaves the anti-sigma factor CalpT, resulting in the release of the sigma factor CalpS, potentiating an anti-viral transcriptional response (29).

**SAVED-CHAT** – this family fuses a cA_3_-binding SAVED domain to a CHAT-family protease which provides immunity via a cascade of proteolytic activity (30).

**NucC** – a hexameric, cA_3_-activated dsDNA nuclease found associated with both CRISPR and CBASS defence (12,31).

**Cam1** – this family has an N-terminal helical transmembrane (TM) domain fused to a C-terminal CARF domain and is activated by cA_4_, resulting in membrane depolarization (32).

**Csx23** – a membrane protein consisting of a tetrameric soluble domain that binds cA_4_, fused to an N-terminal TM helical domain (33).

**CorA** – a TM-domain protein with distant homology to the magnesium channel CorA. This effector is activated by the SAM-AMP signalling molecule and is thought to provide immunity by membrane depolarization (15).

In addition to these ten effector families, further candidate effectors have been implicated in type III CRISPR defence by bioinformatic, guilt-by-association studies (34,35). The overall picture is highly complex and there is clearly more to be discovered. Here, we undertook a systematic analysis of type III CRISPR systems in complete prokaryotic genomes by building a phylogenetic tree for Cas10 followed by characterization of known ancillary effectors, their genomic neighbourhoods and co-occurrence patterns. After characterization of these loci, we turned our attention to loci that showed no known effector proteins but were still likely to produce second messenger molecules due to the presence of a conserved cyclase domain in Cas10. This targeted approach uncovered several potential new classes of type III CRISPR-Cas effectors. One of these, Csm6-2, is confirmed as a novel ribonuclease effector activated by cA_6_.

## Materials and methods

### Data preparation

All complete bacterial and archaeal genome assemblies (both GCA and GCF versions, 76 826 in total) were downloaded from Genbank on 7 September 2023 using NCBI’s Datasets command line client. Bacterial and archaeal genomes were downloaded separately, each genome marked by its respective domain into a separate taxon file and then the datasets merged into one. Genomes were then filtered by the presence of Cas10. First, all proteomes were filtered by protein minimum length of 500 aa to accommodate only functional Cas10 proteins. Then all >500 aa proteins were run against a Cas10 HMM library customized from a previous study (36) with an *E*-value cutoff of 1e-20 using hmmscan from the Hmmer 3.3.2 package (37). The Cas10 HMM library was customized by adding more recent versions of two profiles to make the library compatible with Hmmer 3.3.2: Cas10_0_IIIB (updated using NCBI HMM accession TIGR02577.1) and Cas10_0_IIIA (updated using NCBI HMM accession TIGR02578.1). CRISPR-Cas type I associated Cas10s were removed from the HMM library. The HMM search found 3147 Cas10 proteins, which were then clustered using CD-HIT 4.8.1 (38) with a cutoff (-c) of 0.9 and word size (-n) 5. The clustering step removed most redundancy between GCA and GCF versions of the same genomes. The remaining 902 genomes with unique Cas10 proteins were used in all downstream analyses.

### Characterization of CRISPR-Cas type III loci

We ran CCTyper (36) for all 902 genomes. Loci not designated as type III were excluded from our dataset. Hybrid loci (type III merged with another type III subtype or another CRISPR-Cas type) with more than one *cas10* gene were also removed from the analysis. Remaining hybrids were named after the type III subtype in cases where type III was hybridized with another CRISPR-Cas type. The CCTyper-defined subtype classifications were altered manually in rare cases where there was clearly an incorrect classification.

### Cas10 characterization

The cyclase that generates signal molecules in Cas10 is the PALM2 domain, commonly characterized by the sequence motif GGDD. Manual inspection revealed that while the GGDD motif predominated, sequence variants AGDD, GGED, GGDE, SGDD, DGDD, AGDE, EGDD, KGDD and GEDD were observed in Cas10 sequences present in loci with a known effector protein (and thus likely to be active cyclases). To detect the cyclase domain, HMM profiles were generated by aligning sequences comprising 50 aa N-terminal to and 100 aa C-terminal to the cyclase motif with Muscle 5.1 (-super5 option) (39) and the profiles built using hmmbuild followed by hmmpress in Hmmer 3.3.2 (37). All Cas10s from the type III loci were then queried against these databases with an E-value cutoff of 10^−3^ to determine the presence or absence of the cyclase domain. Finally, to reduce false positives, the literal cyclase motifs listed above were searched for in the positive matches. If no hit against any of these motifs were found, the Cas10 was characterised as not having an active cyclase domain despite a positive HMM hit.

To find nuclease domains in Cas10s, a similar approach was used. The HD sequence motif, the hallmark of the Cas10 nuclease domain, is usually located between 10–35 residues from the N-terminus. From each Cas10 that had the sequence ‘HD’ within the first 50 AA, residues 10–40 were extracted. These sequences were then used to construct HMM profiles as with the cyclase profiles. Each Cas10 was queried against this database with an E-value cutoff of 1e-1. The more relaxed cutoff was used to accommodate the large diversity of the nuclease domain included in the singular HMM profile. Manual inspection was performed to verify the lack of false positives.

A phylogenetic tree of Cas10s was constructed by first aligning the Cas10s with Muscle using the -super5 argument (39). The alignment was used as input for FastTree to create a phylogenetic tree with –wag and –gamma arguments (40). The tree was rooted and visualised using ggtree (41) in R.

### Known effector typing

HMM databases were made from all 10 experimentally characterized type III effectors. Most effector families consisted of several HMM profiles concatenated into one to cover the high sequence diversities. The largest family by number of HMM profiles was Csx1, consisting of 10 HMM profiles. The HMM profiles were refined through an iterative approach, where the HMM profiles for each effector were diversified and adjusted as new variants of a given effector were discovered through manual inspection of the annotated loci. All proteins encoded within the CCTyper operon boundaries ±4 kb were inspected against these profiles, and significant hits to any profile within an effector profile class then counted as an instance of the given effector. In case of hits against multiple effectors, the best-scoring hit by bitscore was chosen. In cases where multiple effectors scored high for given protein sequences, special rules were made to differentiate between effector classes. Csx1 and Cam1 cross-annotations arising from CARF domains present in both effector families were resolved by requiring a transmembrane domain for Cam1 and its absence for Csx1. Transmembrane domains were predicted using the tmhmm.py Python wrapper (https://github.com/dansondergaard/tmhmm.py) for TMHMM (42). Other problematic cross-annotations were resolved by trimming the HMM profiles to exclude common sensory domains (e.g. CARF or SAVED), thus only including the hallmark effector domains. Cross-annotations between effector classes were refined through several runs of the pipeline until no apparent cross-annotations emerged.

Each protein that was determined as an effector was subjected to further characterization by HMM search against the COGs (43), PDB (44) and PFAM (45) databases as well as SAVED and CARF databases from (46). These results are made available as an Excel file (Supplementary Information).

### CorA and its accessory proteins

To further characterize the diversity and genomic neighbourhoods of the recently discovered CorA effector, Diamond (47) databases for the CorA ancillary proteins NrN, DEDD and SAM-lyase were created from homologous protein sequences downloaded from NCBI. Proteins within CorA containing CRISPR-Cas loci were then blasted against this database. A phylogenetic tree of CorA was created by first aligning them with Muscle using the -super5 argument (39) and then creating the tree with FastTree using -wag and -gamma arguments (40). The tree was visualized with ggtree (41) in R and RStudio.

### Identification of new effectors

To find novel effector candidates, CCTyper gene annotations from all type III loci were examined. Any genes that were annotated as ‘Unknown’ by CCTyper or had a poor e-value with any annotation (>1e-07) were flagged as potentially interesting. This list of potential effectors was further refined by analysing their genomic neighbourhoods: if the associated CRISPR type III locus had previously known effectors, the candidate protein was excluded. All proteins that survived these filtering steps were clustered using CD-hit 4.8.1 (38) with a cutoff of 0.4 and word size 2. The representative sequences were then blasted against the proteome database of all loci in the type III CRISPR-Cas collection. The representative proteins were also subjected to HMM search against COGs (43), PDB (44), PFAM (48)and CARF/SAVED databases (46) as well as determination of transmembrane regions using TMHMM (49). Manual inspection of the results revealed several new effector candidates, but also false positives that were not associated with CRISPR-Cas.

The most promising effector candidates were made into HMM profiles by manually blasting them against NCBI’s protein database and creating HMM profiles from the aligned hits. These profiles were used as databases for more sensitive searches against the type III CRISPR-Cas proteomes. Closer examination of loci with candidate effectors also revealed other colocalized proteins that were not picked up by our algorithm due to their significant hits against uncharacterized proteins, such as CasR, in the initial CCTyper search or due to the presence of a previously known effector in the same locus. Such ‘guilt-by-association effector candidates’ were also added to the candidate list upon discovery. This list was then manually curated to remove clear non-CRISPR related genes or diversified versions of known effectors. In the latter case, the HMM libraries used for the known effectors were updated to include profiles made from these diversified homologs, enhancing the performance of our pipeline in subsequent runs. In some cases, the candidate effector HMM profiles cross-annotated proteins that were already annotated with the known effector libraries. Scripts to detect cross-hits between libraries against a single protein were written and the HMM profiles trimmed correspondingly to narrow the hits range for cross-hitting effectors until no cross-hits emerged. Manual inspection was then carried out to verify non-overlapping annotations. Finally, after multiple iterations of the above procedure, the remaining four new candidate effectors were TIR-SAVED, Cam2, Cam3 and Csm6-2.

### Cloning, expression and purification of Csm6-2

A synthetic gene (g-block) encoding *Actinomyces procaprae* Csm6-2, codon optimized for expression in *Escherichia coli* was purchased from Integrated DNA Technologies (IDT), Coralville, USA, and cloned into the pEhisV5Tev vector (50) between the NcoI and BamHI restriction sites. Positive clones were sequenced at Eurofins Genomics, Germany GmbH, to verify the sequence. The pEV5HisTEV-Csm6-2 plasmid was transformed into C43 (DE3) *E. coli* cells. Protein was expressed according to the standard protocol previously described (50). 4 l of culture were induced with 0.4 mM isopropyl-β-d-1-thiogalactoside (IPTG) at an OD_600_ of ∼0.8 and grown overnight at 25°C. Cells were harvested (4000 rpm; Beckman Coulter JLA-8.1 rotor) and resuspended in lysis buffer containing 50 mM Tris–HCl pH 7.5, 0.5 M NaCl, 10 mM imidazole and 10% glycerol, and lysed by sonicating six times 1 min on ice with 1 min rest intervals. Csm6-2 was purified with a 5 ml HisTrapFF column (Cytiva, Marlborough, USA), washed with 5 column volumes (CV) of buffer containing 50 mM Tris–HCl pH 7.5, 0.5 M NaCl, 30 mM imidazole and 10% glycerol, and eluted with a linear gradient of buffer containing 50 mM Tris–HCl pH 7.5, 0.5 M NaCl, 0.5 M imidazole and 10% glycerol across 15 CV on an AKTA purifier (Cytiva). Protein containing fractions were concentrated and the 8-his affinity tag was removed by incubation of protein with Tobacco Etch Virus (TEV) protease (10:1) overnight at room temperature. Cleaved Csm6-2 was separated from TEV by repeating the immobilised metal affinity chromatography step and the unbound fraction collected. Size exclusion chromatography was used to further purify Csm6-2, with the protein eluted isocratically with buffer containing 20 mM Tris–HCl pH 7.5, 250 mM NaCl. The protein was concentrated using a centrifugal concentrator, aliquoted and stored frozen at –70°C.

### RNAse activity of Csm6-2 effector

The RNase activity and the activating signal molecule for Csm6-2 were determined using an RNAse Alert assay (IDT). Csm6-2 (100 nM) was incubated in a 25 μl reaction (Tris–HCl 20 mM pH 7.8, NaCl 100 mM, MgCl 10 mM, RNAse Alert substrate 100 nM) with cA_3_, cA_4_ or cA_6_ (1 μM) in triplicates at 37°C. The cA_4_ activated ribonuclease TTHB144 (100 nM) was used as a positive control (21). Fluorescence was measured at 485/520 nm wavelength every 15 s using a plate reader over 1.5 h and the resulting data visualized with ggplot (51) in R (v4.3.0; R Core Team 2023).

To visualize Csm6-2 ribonuclease activity by gel electrophoresis, Csm6-2 (10 nM) was incubated with a 60 nt ssRNA substrate (5′-AUUGAAAGACCAUACCCAACUUCUAACAACGUCGUUCUUAACAACGGAUUAAUCCCAAAA) with a 5′-fluorescein amidite (FAM) label (400 nM) and optionally cA_3_, cA_4_ or cA_6_ (1 μM) in a 14 μl reaction (Tris–HCl 20 mM pH 7.8, NaCl 100 mM, MgCl 10 mM). After incubating for 1 h at 37°C, RNA was denatured for 2 min at 95 °C and mixed with 100% formamide (1:1) on ice. 20 μl of the samples were run on a 20% urea–PAGE gel at 30 W with 45 °C temperature limit for 1 h 15 min. The gel was scanned with a Typhoon FLA 7000 imager (GE Healthcare) using wavelength 532 nm.

### Protein structure prediction

Protein structures were predicted using Alphafold2 (AF2) as implemented in the Colabfold server (52,53). Trans-membrane regions were predicted using DeepTMHMM (54). Raw output and statistics for prediction accuracy are shown.

## Results

### A phylogenetic tree of the Cas10 protein

**Figure 1.**
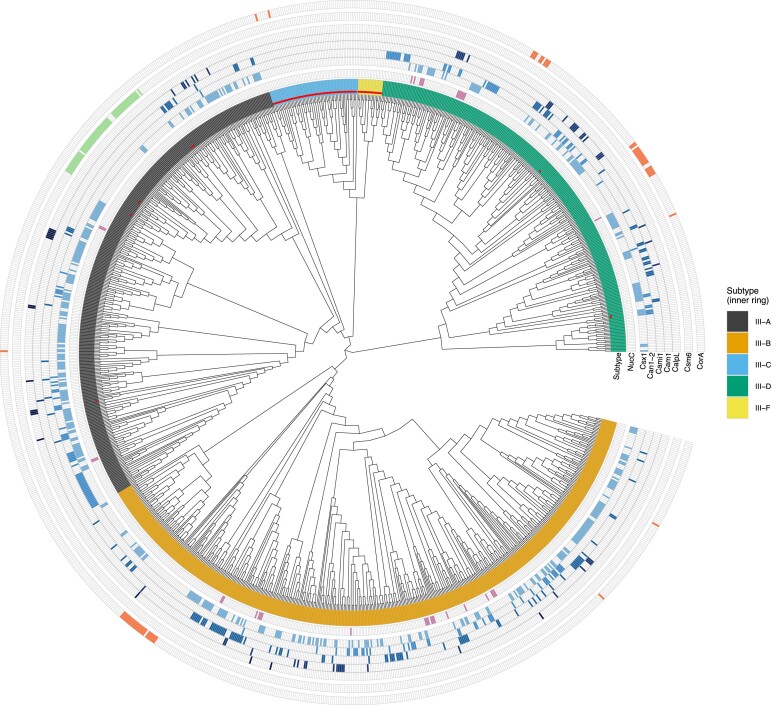
Phylogenetic tree and associated effectors for Cas10. The inner multi-coloured ring shows the subtype of the associated type III CRISPR-Cas locus. The next rings show the presence or absence of eight of the most common known effectors. Effectors are divided in ring groups by their associated signal molecule, so that cA_3_ (NucC), cA_4_ (from Csx1 to CalpL), cA_6_ (Csm6) and SAM-AMP (CorA) associated effectors are in their respective groups separated by gaps between rings. Red dots indicate Cas10s with no detectable cyclase domain.

To analyse our dataset, we generated a phylogenetic tree of Cas10s annotated with cyclase domains and associated effector proteins (Figure 1). Our dataset comprises 1113 type III CRISPR loci of which 437 (39%) contain a recognizable HD nuclease domain in the associated Cas10 protein (Supplementary Figure S1). HD domains are most common in type III-A systems (65%) and least common in type III-D systems (3%), suggesting that type III-D functions primarily through cOA signalling. Overall, a cyclase domain is present in 1028 (92%) Cas10s while 34% have both the nuclease and the cyclase domain, confirming previous estimates (55). Subtypes III-A and III-B are quite heterogeneous, with HD domains frequently present and cyclase domains near-ubiquitous. Cyclase active sites are absent from type III-C loci, which corresponds to a lack of known effectors for this subtype (Figure 1). Half of the type III-C systems have a recognizable HD nuclease domain, suggesting that they may provide antiviral immunity without recourse to cOA signalling. This is also true for the type III-F systems in the dataset, which generally have HD nuclease but which all lack cyclase motifs and effectors.

### Distribution of characterized type III CRISPR ancillary effectors

**Figure 2.**
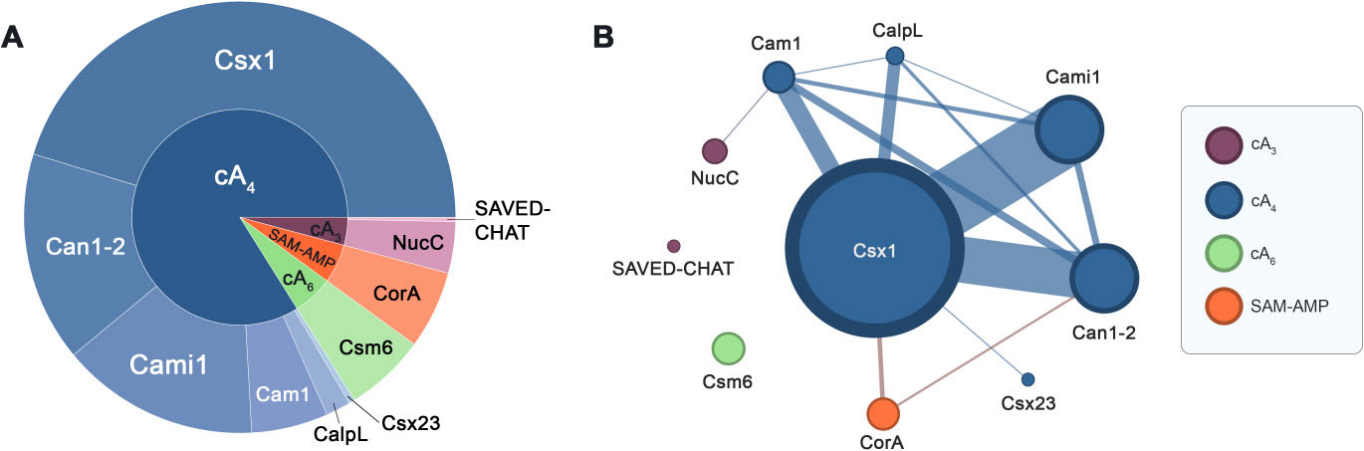
Abundance and co-occurrences of type III CRISPR effectors. (**A**) Pie chart of known effectors and activation signals. The outer ring shows the proportion of each effector in the dataset and the inner ring indicates the activator. (**B**) Network plot of known effectors. Sphere size is proportional to the total count of each effector in our dataset. Lines between effectors indicate co-occurrence of the two effectors within the same loci, with line thickness proportional to the number of co-occurrences. Nodes are coloured by their presumed activating signal molecules using the same colour scheme as in panel A. Network visualised using Gephi (https://gephi.org/).

We mapped and quantified the occurrence of each of the 10 known effectors in the CRISPR loci in our dataset (Figure 2). We took the decision to disregard ancillary proteins that were likely involved in regulation of the immune response, including predicted transcription factors such as Csa3 (56) and WYL (57), along with ring nucleases (58–60). These will be analysed in a future study. In total, 908 effectors were identified across the 1113 loci. The most common was Csx1, present in 411 loci, followed by Can1-2 (143 loci), Cami1 and Cam1 (135 and 52 loci, respectively). CalpL (17 loci) and Csx23 (4 loci) complete the set of cA_4_ activated effectors. In our dataset, with the assumption that all members of the effector families defined here share the same activator, we calculate that 84% of known effectors are cA_4_-activated, making this the predominant second messenger in type III CRISPR signalling. In contrast, cA_6_ is only known to activate Csm6 proteins, which are present in 55 loci and found in a narrow phylogenetic area of the tree in type III-A loci (Figure 1). The cA_3_ activated NucC effector is broadly scattered in the tree in 35 loci. As noted previously (55), there are rare examples where NucC is fused to the Cas10 subunit. This is the case in the *Virgibacillus pantothenticus* genome, where a standalone *nucC* gene is adjacent to the *nucC-cas10* gene. This arrangement may allow NucC to hexamerize while associated with the type III-D complex. The recently described SAVED-CHAT effector (30) is quite rare, present in only three loci. Finally, the SAM-AMP activated CorA effector is found in 53 loci, in three main clusters in the tree, as described previously. Network analysis (Figure 2B) indicates that cA_4_-activated effectors co-occur in loci relatively frequently – this will be explored in greater detail in the following section.

To facilitate exploration of the data by third parties, we provide an interactive web portal that allows visualization and filtering of the annotated loci in this study. The website is available at https://vihoikka.github.io/type_iii_crispr_browser.

### New candidate type III CRISPR ancillary effectors

**Figure 3.**
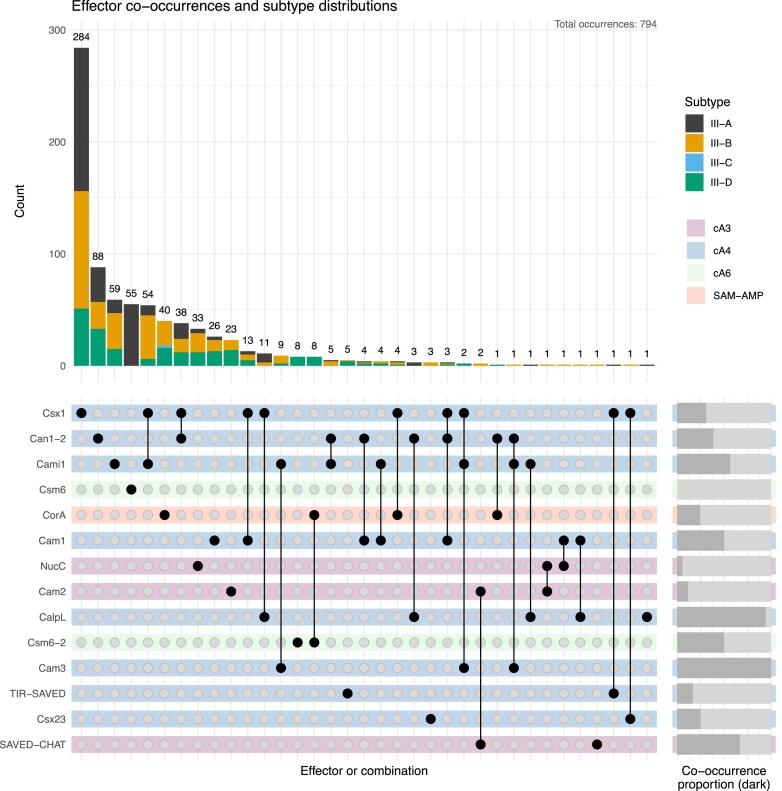
Upset plot of type III CRISPR effector co-occurrences. The stacked bar chart on the top visualizes the abundance of each effector and their respective CRISPR-Cas subtypes. The effector configuration for each stacked bar is displayed by the dot matrix underneath the bars. For example, Csx1 is present 284 times on its own and 54 times with Cami1. The light backgrounds behind the configuration dots indicate the presumed signal molecule associated with the effectors as shown in the legend. The co-occurrence proportion chart on the right side shows how often an effector is co-occurring: a completely dark chart indicates 100% co-occurrence (e.g. Cam3) while a completely light chart indicates that an effector occurs purely on its own (e.g. Csm6).

Having identified all instances of the 10 characterized type III CRISPR ancillary effectors in our dataset, we further examined the loci which fulfilled the following conditions: (a) no known effector present; (b) Cas10 has a clear cyclase domain and lacks an HD domain. We reasoned that examination of the genes present in these loci might reveal new effector families, and this proved to be the case, resulting in identification of four new candidate effectors. These are described in turn below and an Upset plot showing their distribution and co-occurrence with the ten characterized effectors is shown in Figure 3.

#### TIR-SAVED: a moonlighting CBASS effector

The TIR-SAVED effector was first experimentally described in the context of CBASS systems, where cA_3_ binding by the SAVED domain results in the formation of an extended helical filament that allows self-association and activation of the TIR domain, leading to NAD+ degradation (61). This effector provided antiviral defence when used to replace the cognate Csm6 effector in a type III CRISPR system (61), so it is perhaps not surprising that TIR-SAVED effectors are detected in seven loci, corresponding to CRISPR types III-A, III-B and III-D (Figure 3). SAVED domains have a wide range of activators from cyclic di-, tri- and tetranucleotides (29,61,62). In *Halocatena*sp.*RDMS1*, TIR-SAVED is present in a locus that includes a Csx1 effector. We therefore tentatively suggest that the CRISPR-specific TIR-SAVED may be activated by cA_4_. Recently, CARF-TIR effectors have been detected in some type III CRISPR loci (63), and SAVED-TIR proteins have been identified in a large-scale analysis of CARF and SAVED proteins (46).

#### CRISPR-associated membrane protein 2 (Cam2)

This CRISPR-associated protein consists of a predicted N-terminal TM helical domain of variable length and a C-terminal domain with clear structural homology to the REC domain of Response Regulator (RR) proteins. Canonical REC domains are typically phosphorylated by a histidine kinase partner on a conserved aspartate residue, eliciting structural changes and a downstream response (64). Given the lack of an associated histidine kinase, canonical function via phosphorylation seems unlikely. REC domains display a lot of functional plasticity and can also be activated by ligand binding (64). For example the transcription factor JadR1 REC domain binds the antibiotic JdB, disrupting DNA binding (65). Our working hypothesis is that the REC domain of Cam2 binds a cOA signalling molecule, given its association with type III CRISPR systems.

**Figure 4.**
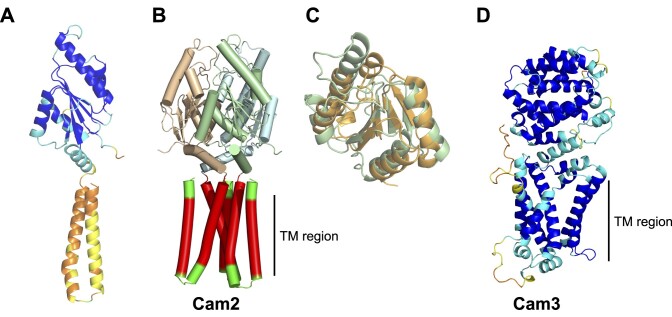
AF2 models of the Cam2 and Cam3 effectors (**A**) AF2 model of Cam2 monomer, showing the N-terminal predicted TM helical domain and C-terminal response regulator (REC) domain, coloured by AF2 pLDDT prediction score. (**B**) Trimeric model for Cam2, coloured by subunit, with the TM region shown in red. (**C**) Structural overlay of the REC domain of Cam2 (green) with a REC domain of a response regulator (orange) (PDB 3lua; Dali score 9.3, RMSD 2.3 Å over 99 residues (66)). (**D**) AF2 model of a Cam3 monomer, showing the N-terminal predicted TM helical domain and C-terminal soluble domain, coloured by AF2 pLDDT prediction score. AF2 confidence statistics are shown in Supplementary Figure S1.

The *cam2* gene is found in 26 CRISPR loci in the dataset. In one case it is adjacent to a gene encoding NucC and in 2 cases next to a gene predicted to encode a SAVED-CHAT protein (Figure 3). Since NucC is activated by cA_3_ (31) and SAVED-CHAT proteins found in type III CRISPR and CBASS systems are also cA_3_ activated (30), we predict that the Cam2 family are also cA_3_ activated effectors. We have modelled Cam2 as a trimer based on the assumption that it binds the cA_3_ activator, which has 3-fold symmetry (Figure 4; Supplementary figure S2), but this requires confirmation. We predict that cA_3_ binding to the REC domain results in structural changes in the TM domain that could result in disruption of the membrane integrity, analogous to the mechanism of the Csx23 and Cam1 effectors (32,33). If this prediction is correct, Cam2 represents a novel class of cA_3_ binding effector and is a priority for further study.

#### CRISPR-associated membrane protein 3 (Cam3)

Cam3 is encoded in 12 type III-B CRISPR loci. It is always found immediately downstream of the gene encoding Cami1, suggesting they may function together to provide defence (Figure 3). AF2 predicts a compact N-terminal helix-rich soluble domain and a six-helix bundle, which corresponds with the prediction of 6 TM helices by DeepTMM (54) (Figure 4D; Supplementary figure S2). Dali searches (66) yield only hits to a portion of the predicted TM helical bundle, and there is little sequence conservation in the predicted soluble domain. The likely function of Cam3 thus remains enigmatic and requires follow-up study. Given its universal association with the Cami1, Cam3 may be an accessory protein rather than an effector activated by cyclic nucleotide binding.

#### Csm6-2: a fused, monomeric CARF-HEPN-CARF-HEPN effector

**Figure 5.**
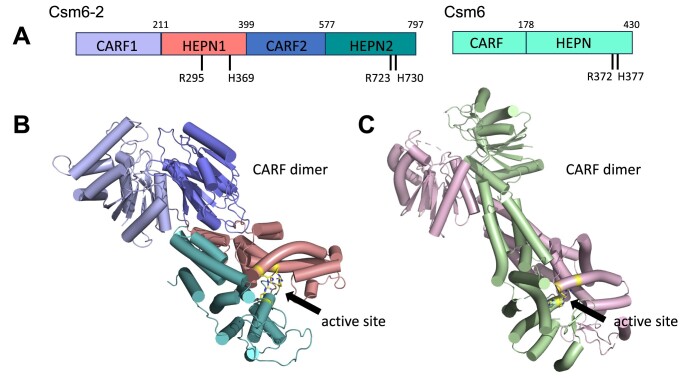
Domain organisation and AF2 structure prediction for the Csm6-2 effector (**A**) Comparative domain organisation of Csm6-2 from *A. procaprae* (WP_136192673.1) and *Enterococcus italicus* Csm6 (24). The active site residues of the HEPN domains are indicated. (**B**) AF2 model of *A. procaprae* Csm6-2, domains coloured as in (A). (**C**) Structure of canonical Csm6 from *E. italicus* (PDB 6TUG), subunits coloured in green and pink. Side chains of active site R and H residues are shown in yellow.

A novel ribonuclease, Csm6-2, with a domain organization consisting of CARF-HEPN-CARF-HEPN in a single fused polypeptide of ∼795 amino acids was observed in 16 type III-D loci (Figure 3). The signature R(X_4-6_)H motif of the HEPN ribonuclease domain is observed in the HEPN2 domain, whilst these two residues are separated in the primary sequence by 73 amino acids in the HEPN1 domain (Figure 5A). The AF2 model of Csm6-2 highlights the structural similarity with canonical Csm6 dimers and positions the two nuclease active sites similarly to those in canonical, dimeric Csm6 proteins (Figure 5B,C; Supplementary figure S2). Csm6-2 presumably arose from a Csm6 ancestor by gene duplication, fusion and divergence, analogous to the relationship between Can1 and Can2 (25,26).

**Figure 6.**
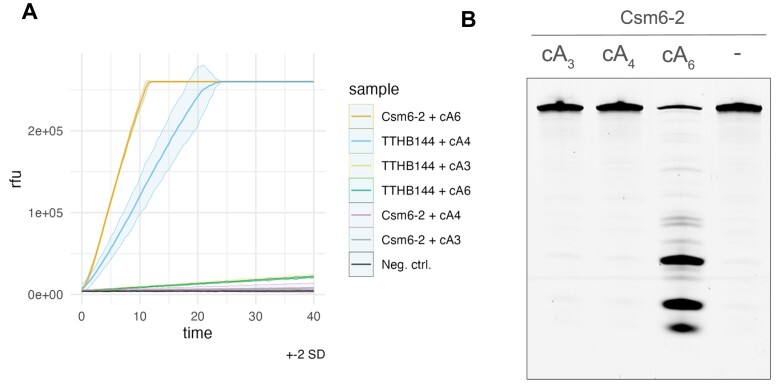
Csm6-2 is activated by cA_6_. (**A**) RNAse alert assay shows fluorescence resulting from RNA cleavage when Csm6-2 is incubated with cA_6_ or the control effector TTHB114 is incubated with cA_4_. Solid lines are means of three replicates and the surrounding tinted region shows the ± 2 standard deviation range. (**B**) Csm6-2 cleaved a 5′-FAM-ssRNA after 1 h incubation in the presence of cA_6_.

To confirm our bioinformatic predictions, we cloned and purified the *Actinomyces procaprae* Csm6-2 homologue to test its RNAse activity *in vitro*. As CARF domains are expected to bind cyclic oligoadenylates, we incubated Csm6-2 with cA_3_, cA_4_ or cA_6_ in an RNAse Alert assay and measured fluorescence released through RNA cleavage (Figure 6A). Csm6-2 RNAse activity was triggered only by cA_6_. As a positive control, we tested the Csx1 family nuclease TTHB144, which is induced by cA_4_ (21). To confirm this result and visualise RNA cleavage sites, we incubated Csm6-2 in the presence of cA_3_, cA_4_ or cA_6_ and a 60 nt ssRNA substrate labeled with a 5′-fluorescein amidite (FAM) label. Upon separation of the RNA fragments by denaturing PAGE, fluorimetry showed cleavage only in the cA_6_-containing sample (Figure 6B). The range of RNA products is typical of HEPN ribonucleases with relaxed sequence specificity (21,23,24). Taken together, these results show that Csm6-2 is a highly divergent member of the Csm6 ribonuclease family, activated by cA_6_. As canonical Csm6 enzymes have applications in CRISPR-based diagnostics (67,68), further characterization of this enzyme is warranted.

### Inter-locus signalling between type III loci?

We found 133 loci with no known effectors or credible candidates for new ones, while still coding for a nuclease-deficient Cas10 with a cyclase domain. One possible explanation for the lack of effectors in these loci is that signal generation in one locus may lead to the activation of effectors encoded by another locus in the genome. In *trans* sharing of components between CRISPR-Cas loci has indeed been observed in spacer acquisition (69), interference (70) and crRNA processing (71). To investigate if effector-lacking loci are more likely to be associated with other type III loci in the same genome, we created a generalized linear model with effector presence as the response variable and having multiple type III loci in the genome as the explanatory variable. According to this model, when a locus lacks effectors, its associated genome is 2.57 times more likely to contain multiple type III loci compared to a locus with one or more effectors (*P* = 4.71e-12, *Z* = 6.914, binomial GLM). This observation suggests that some effector-lacking yet cyclase-positive type III loci may have adapted to activating effectors coded elsewhere in the genome.

### Co-occurrence patterns of type III CRISPR effectors

Although cooperation between multiple type III CRISPR effectors in a single locus has not been studied in detail, co-occurrence is a relatively common situation in our dataset, at least for cA_4_-activated effectors (Figure 3). For example, the most abundant effector in our dataset, Csx1, is found on its own in 284 loci and in combination with others in 127 loci (31% co-occurrence). Cam1 and Cami1 are found co-occurring with other effectors in around 50% of cases whilst CalpL is seldom found alone. These are all examples where two effectors, each activated the same cA_4_ species, are present in one locus and presumably provide broad defence by targeting two different biomolecules simultaneously to slow down viral infection. Csx1 co-occurs at least once with each of the other known or predicted cA_4_-activated effectors in the dataset, suggestive of considerable flexibility in effector cooperation. The co-occurrence of 3 effectors is rare, but we detected 3 examples where Csx1, Can1-2 and Cam1 were all present in a locus. Restricting the analysis to type III CRISPR loci in the archaea, subtly different patterns of occurrence and co-occurrence are observed (Supplementary figure S3), with Csx1 the dominant effector and no examples of cA_3_ or cA_6_-activated effectors present in archaeal genomes. CalpL, which signals via a sigma factor in a mechanism specific for the bacterial transcription machinery (29) is also absent, as expected.

By contrast, co-occurrence of effectors using different cOA signals was very rare; for example, the cA_6_-activated Csm6 is never found alongside a cA_4_-activated effector. The sole example in our dataset is where a cA_3_-activated NucC enzyme co-occurs with the cA_4_-activated Cam1 effector. As a general rule then, we can hypothesize that individual CRISPR loci tend to use one cOA species in antiviral defence, even though they can generate multiple cOA species both *in vitro* and *in vivo* (6,11–14). The exception to this rule appears to be CorA, which we turn to now.

### Diverse activating molecules for the CorA effector?

**Figure 7.**
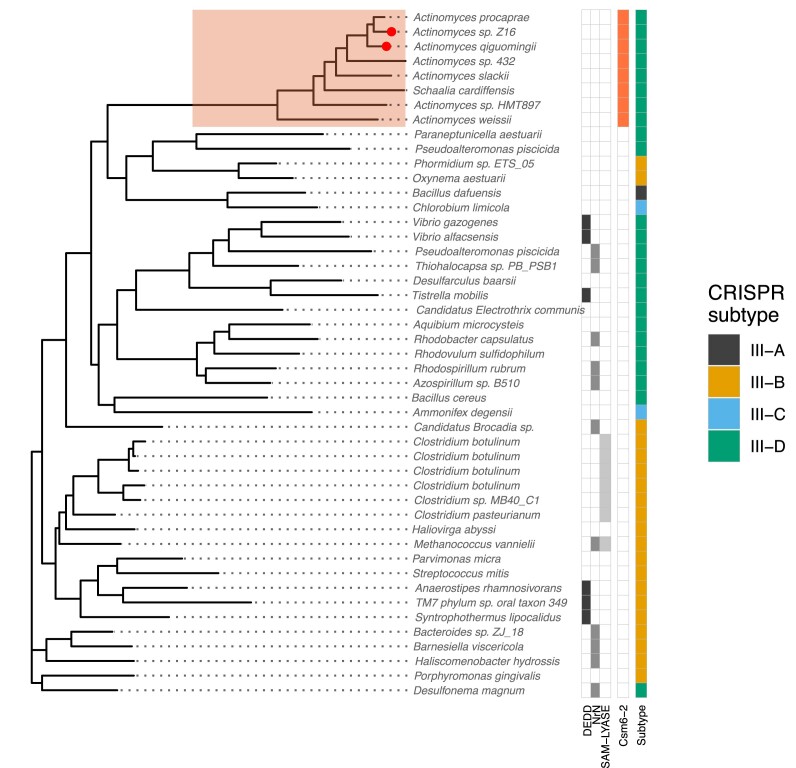
Phylogenetic tree of CorA family effectors. CorA-associated ancillary proteins (DEDD, NrN and SAM-Lyase) are shown for each locus along with the cA_6_ activated Csm6-2 proteins, and CRISPR-Cas subtype. Based on Csm6-2 association, we predict the highlighted clade to be a cA_6_ activated CorA subclass. Two CorA/Csm6-2 fusions are marked with red dots.

The CorA effector is found in three main clusters of type III loci (Figure 1) (15), one of which is also associated with the newly discovered Csm6-2 effector (Figure 3). The cluster associated with type III-B systems such as those in *Bacteroides fragilis* is activated by SAM-AMP, which is degraded by associated NrN or DEDD phosphodiesterases, or lyases (15). We therefore investigated the CorA phylogenetic tree and its co-occurrence with SAM-AMP degrading enzymes and the Csm6-2 effector in more detail (Figure 7). Most CorA proteins are clearly associated with enzymes that degrade SAM-AMP, suggesting that this molecule is the relevant activator. However, a divergent clade of CorA proteins found in the *Actinomyces* lacks these degrading enzymes. Instead, this clade is associated with the Csm6-2 effector. In two cases in this clade, CorA has Csm6-2 fused at the C-terminus of the protein. These observations lead us to speculate that *Actinomyces* CorA effectors are activated by cA_6_, rather than SAM-AMP, as it is hard to envisage that the Cas10 cyclase in the locus can make such divergent nucleotide products as SAM-AMP and cA_6_ in the same active site. In line with this hypothesis, signatures of coevolution between Cas10 and CorA have been observed previously through correlation of their phylogenetic trees (35).

## Discussion

Type III CRISPR systems, which can ‘outsource’ defence to ancillary effector proteins controlled by Cas10-derived nucleotide second messengers, are by far the most diverse of all CRISPR subtypes. New effector proteins are being identified and characterized at an accelerating rate. In this study, we aimed to characterize all type III CRISPR loci in completed genomes in NCBI, allowing us to derive some paradigms, suggest some hypotheses and predict new families of effectors.

Firstly, considering Cas10 itself, we find that cyclase activity (92% predicted occurrence) is much more common than HD-nuclease activity (39%), while around one third of Cas10 enzymes are predicted to harbour both activities. These numbers are broadly similar to a previous study of Cas10 that included metagenomic sequences (55). Turning to ancillary effectors, cA_4_ activated proteins predominate and can co-exist in CRISPR loci in many different combinations, providing the opportunity to target multiple biomolecules simultaneously in response to Cas10 activation. However, loci with a sole effector are still in the majority, perhaps reflecting a trade-off between defence and toxicity. It is thought provoking that there are almost no examples where one CRISPR locus activates effectors with different signalling molecules. For example, Csm6 (cA_6_ activated) is never found with any cA_4_ activated effector, despite the observation that individual Cas10s can function *in vivo* with effectors activated by different cOA species (11,33). One might assume signalling via two different activators would be beneficial to combat viruses with the ability to degrade cA_4_ using ring nucleases, for example (9). One possibility is that type III CRISPR systems in their natural state cannot easily make more than one activator at the concentrations required for antiviral defence.

Our analysis highlights the CorA effector as an interesting outlier. One family of CorA proteins has been shown to be activated by the molecule SAM-AMP – generated by a specialized Cas10 enzyme that can bind *S*-adenosyl methionine (15). However, the co-occurrence and fusion of some CorA effectors with the newly described, cA_6_ activated Csm6-2 enzyme raises the prospect that there are different families of CorA proteins activated by different molecules. This is not wholly unprecedented if one considers that CARF domains have the ability to bind either cA_4_ or cA_6_, but clearly requires experimental follow up. Currently, the lack of structural information on the cytoplasmic domain of CorA is a limiting factor for our understanding, albeit one that is not likely to persist for long.

The recent studies of TM effectors CorA (15), Cam1 (32) and Csx23 (33) highlight the diversity of type III CRISPR ancillary proteins. These examples demonstrate that signalling nucleotides generated for anti-viral defence can be detected by a wide range of cytoplasmic sensing domains, beyond the canonical CARF and SAVED superfamily. This is also exemplified by the Cap15 effector of CBASS defence, which uses a β-barrel domain to bind cyclic nucleotides (72). In this regard, the discovery of Cam2 as a novel proposed TM effector is particularly interesting, as the protein appears to use a Response Regulator (REC) domain for nucleotide sensing—a ubiquitous signal transduction domain that has not previously been associated with nucleotide sensing.

In conclusion, we hope that this analysis, together with the provision of an easily searchable database for type III CRISPR loci, will stimulate further research by the community. We have not considered genomes marked as incomplete, have excluded loci with Cas10 length <500 residues and have not exhaustively tracked down every divergent Csx1 family member. Analysis of transcriptional regulators and ring nucleases, which are frequently present in CRISPR loci, will be topics of future studies.

## Supplementary Material

gkae462_Supplemental_Files

## Data Availability

The Snakemake pipeline that reproduces the analysis is available at https://github.com/vihoikka/hoikkala_etal_typeIII_effectors and at Figshare under the DOI 10.6084/m9.figshare.25451944. This repository also contains the custom HMM profiles for known and new effectors, and R scripts for generating the figures. The interactive website for browsing the data described in this manuscript is available at https://vihoikka.github.io/type_iii_crispr_browser/ or a static .zip file at Figshare under the DOI 10.6084/m9.figshare.25451974.
