# Supplementary material for: Bioinformatic analysis of type III CRISPR systems reveals key properties and new effector families
Source: Nucleic Acids Res. 2024 May 29;52(12):7129–41. doi: 10.1093/nar/gkae462 (PMC11229360; doi:10.1093/nar/gkae462)
Supplement: gkae462_Supplemental_Files [file gkae462_supplemental_files.zip › Supplementary Figures.pdf]

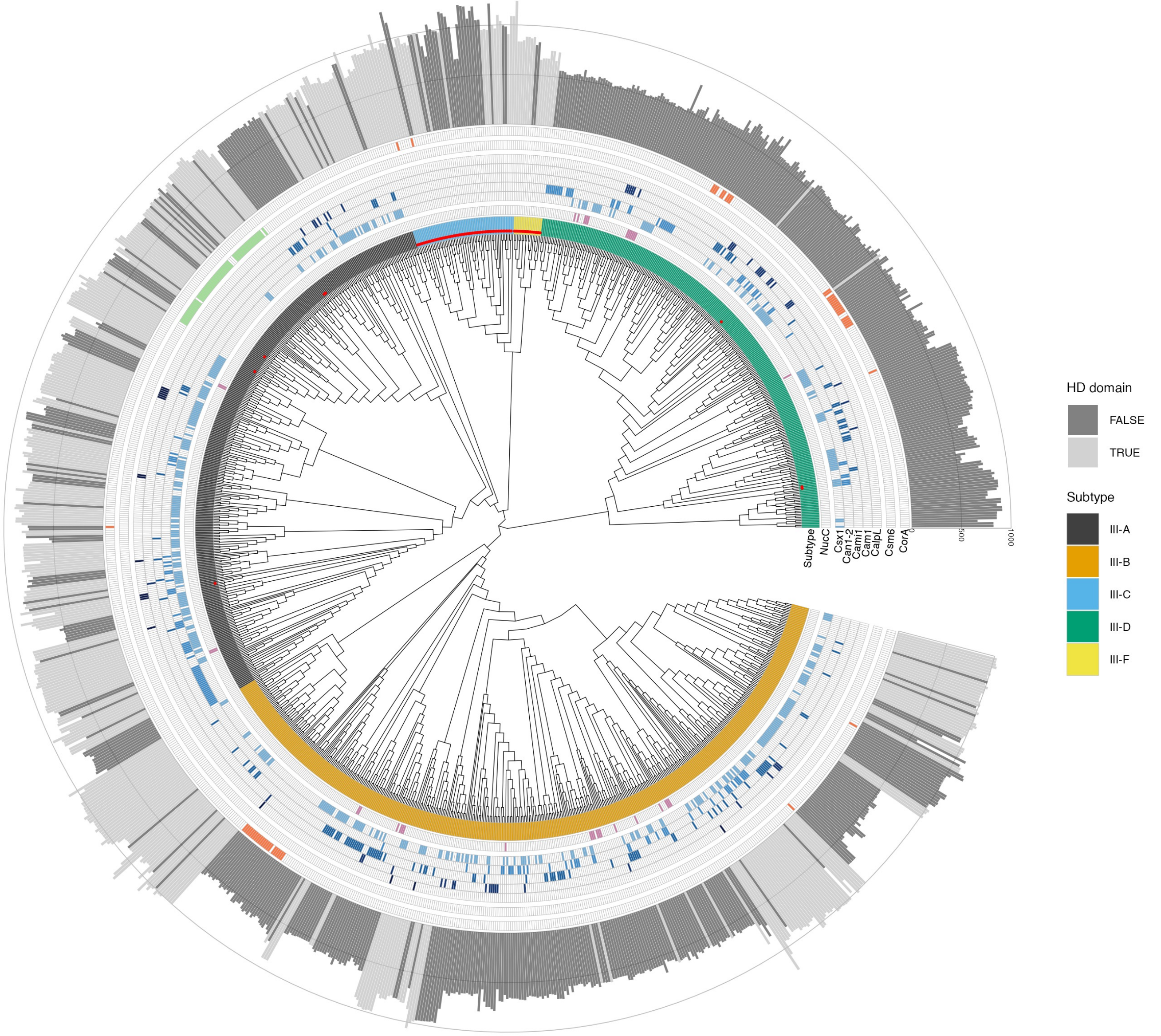

Supplementary Figure 1. Phylogenetic tree of Cas10 homologs and associated effector proteins with additional annotations as compared to main text Figure 1. The inner circle shows the CRISPR-Cas subtype associated with each Cas10 homolog, red dots indicating the absence of a cyclase domain. The next rings show the presence or absence of eight of the most common known effectors. The outmost bar chart illustrates the length of the Cas10 proteins, with scale lines at 500 and 1000 aa. The bars are shaded to represent the presence (light grey) or absence (dark grey) of a nuclease (HD) motif.

A. Cam2 trimer (pLDDT=76.8; pTM=0.718; ipTM=0.695)

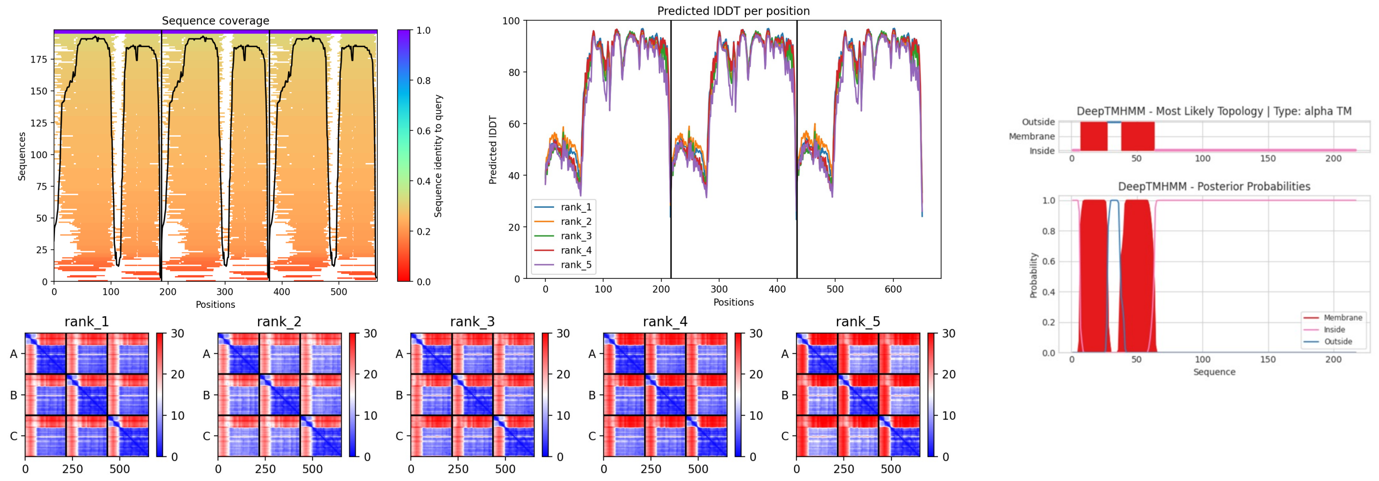

B. Cam3 monomer (pLDDT=90.2; pTM=0.873)

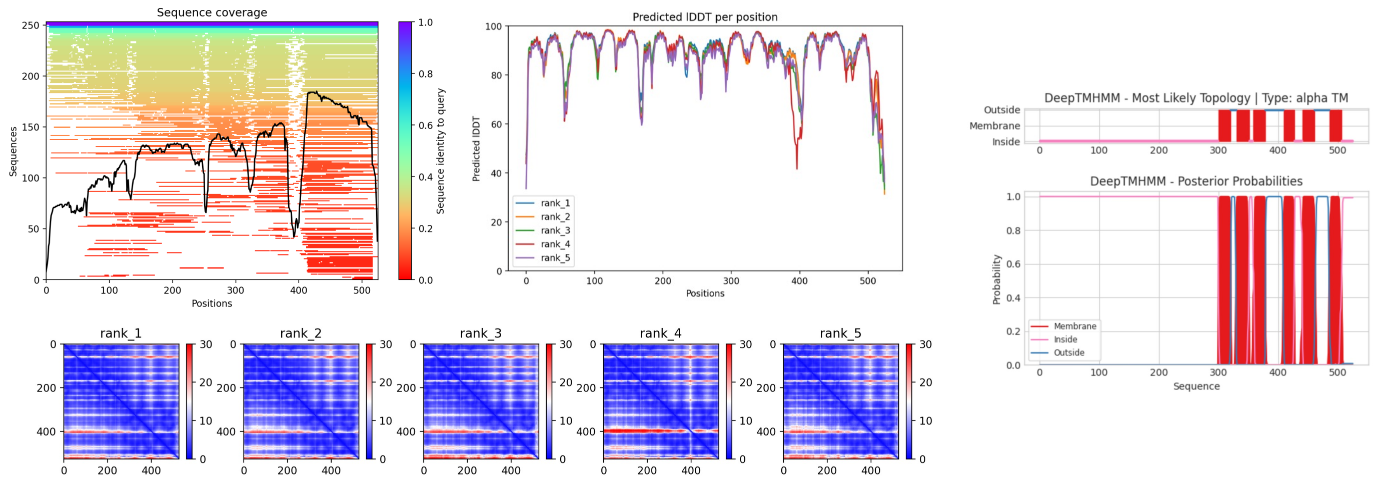

C. Csm6-2 monomer (pLDDT=87.8; pTM=0.881)

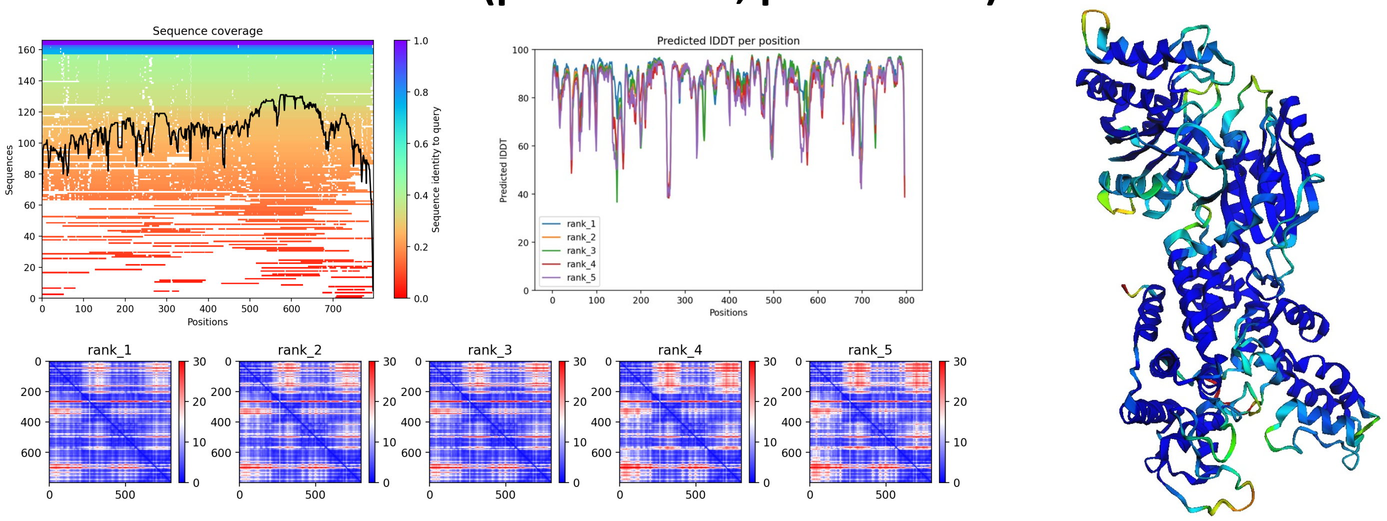

Supplementary Figure 2. Confidence metrics associated with predicted structures of Cam2, Cam3 and Can3. For each protein, the upper left plot shows alignment coverage during the MMseqs2 homology search step. The upper right plot shows the predicted local distance difference test (pLDDT), and the lower plots show the predicted aligned error (PAE). For Cam2 and Cam3, the predicted topology and trans-membrane regions output from DeepTHMM are shown on the far right. For Can3, the AF2 predicted structure coloured by pLDDT is shown on the far right. Predicted template modelling (pTM) scores are shown, along with interface pTM (ipTM) score for the Cam3 trimer model. A dimeric model for Cam2 yielded poorer quality scores than the trimeric model.

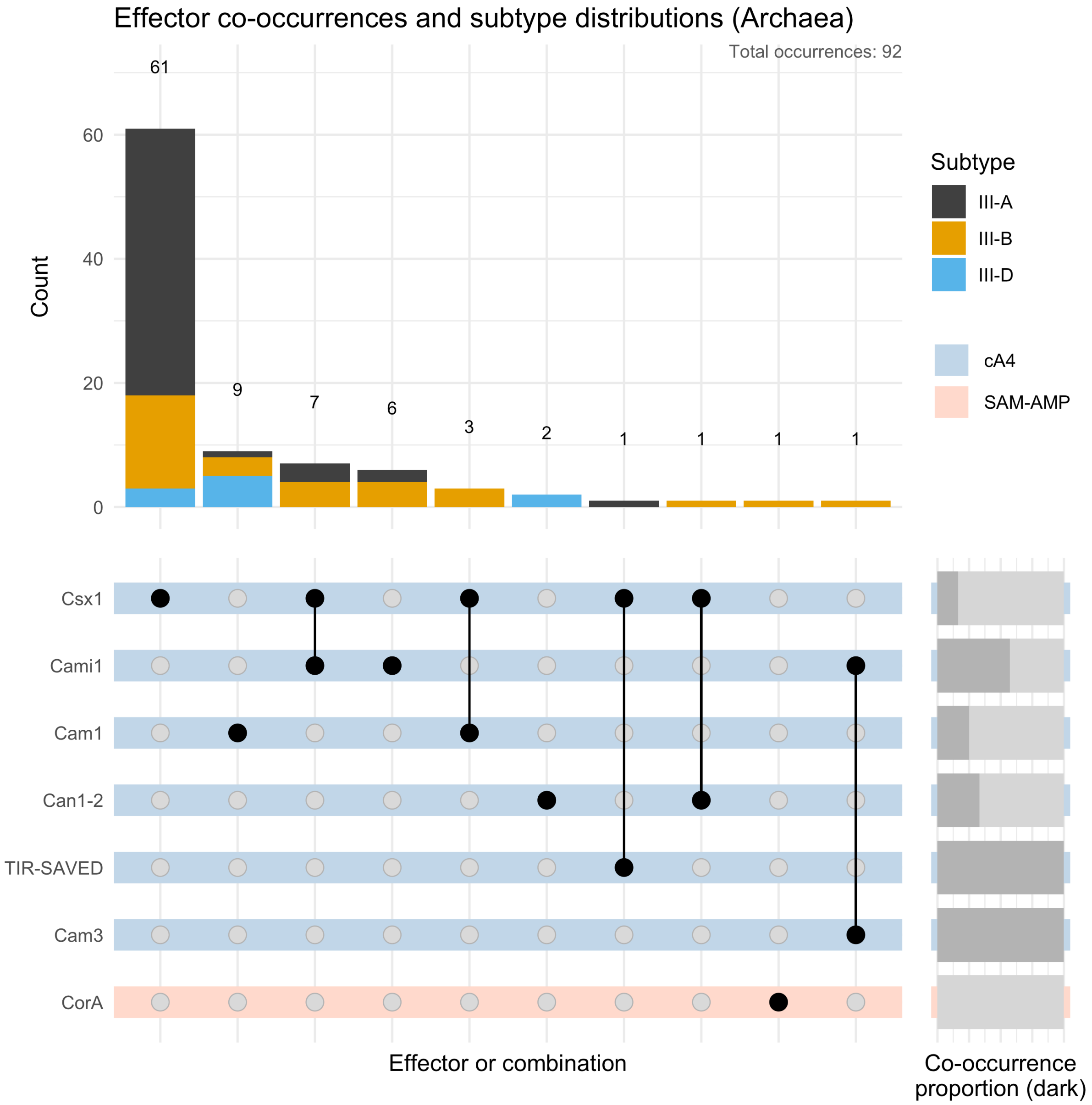

Supplementary Figure 3. Upset plot of type III CRISPR effector co-occurrences in archaea. The stacked bar chart on the top visualises the abundance of each effector and their respective CRISPR-Cas subtypes. The effector configuration for each stacked bar is displayed by the dot matrix underneath the bars. The light backgrounds behind the configuration dots indicate the presumed signal molecule associated with the effectors as shown in the legend. The co-occurrence proportion chart on the right side shows how often an effector is co-occurring: a completely dark chart indicates 100% co-occurrence while a completely light chart indicates that an effector occurs purely on its own.
